# COVID-19 associated Pulmonary Aspergillosis in Patients Admitted to the Intensive Care Unit: Impact of Antifungal Prophylaxis

**DOI:** 10.1007/s11046-023-00809-y

**Published:** 2024-01-13

**Authors:** Jonas Frost, Maximilian Gornicec, Alexander C. Reisinger, Philipp Eller, Martin Hoenigl, Juergen Prattes

**Affiliations:** 1 Division of Infectious Diseases, Department of Internal Medicine, Medical University of Graz, ECMM Excellence Center, Graz, Austria; 2 Intensive Care Unit, Department of Internal Medicine, Medical University Graz, Graz, Austria; 3 BioTechMed Graz, Graz, Austria

**Keywords:** COVID-19-associated pulmonary aspergillosis, Prophylaxis, Posaconazole, Intensive care unit, Respiratory failure

## Abstract

Early after the beginning of the coronavirus disease 2019 (COVID-19)-pandemic, it was observed that critically ill patients in the intensive care unit (ICU) were susceptible to developing secondary fungal infections, particularly COVID-19 associated pulmonary aspergillosis (CAPA). Here we report our local experience on the impact of mold active antifungal prophylaxis on CAPA occurrence in critically ill COVID-19 patients. This is a monocentric, prospective cohort study including all consecutive patients with COVID-19 associated acute respiratory failure who were admitted to our local medical ICU. Based on the treating physician’s discretion, patients may have received antifungal prophylaxis or not. All patients were retrospectively characterized as having CAPA according to the 2020 ECMM/ISHAM consensus definitions. Seventy-seven patients were admitted to our medical ICU during April 2020 and May 2021 and included in the study. The majority of patients received invasive-mechanical ventilation (61%). Fifty-three patients (68.8%) received posaconazole prophylaxis. Six cases of probable CAPA were diagnosed within clinical routine management. All six cases were diagnosed in the non-prophylaxis group. The incidence of CAPA in the overall study cohort was 0.57 events per 100 ICU days and 2.20 events per 100 ICU days in the non-prophylaxis group. No difference of cumulative 84-days survival could be observed between the two groups (*p* = 0.115). In this monocentric cohort, application of posaconazole prophylaxis in patients with COVID-19 associated respiratory failure did significantly reduce the rate of CAPA.

**Supplementary Information:**

The online version contains supplementary material available at 10.1007/s11046-023-00809-y.

## Introduction

Recent studies have highlighted that severe respiratory viral infections such as influenza or coronavirus disease 2019 (COVID-19) pose a risk for secondary fungal infections in critically ill patients [1]. In consequence, influenza-associated pulmonary aspergillosis (IAPA) has been recognized as a new entity that affects immunocompromised as well as non-immunocompromised critically ill patients [2]. In line, a severe course of COVID-19, caused by severe acute respiratory syndrome coronavirus 2 (SARS-CoV-2), may cause respiratory failure and admission to intensive care unit (ICU), complicated by secondary bacterial and/or fungal infections, particularly CAPA [3–5]. The pathogenesis of CAPA is driven by several factors. First, SARS-CoV-2 infections leads to cellular damage in the respiratory epithelium, which is usually considered the first line of defense against fungal infections [6]. Damaged epithelium is associated with reduced ciliary clearance of inhaled fungal spores and altered direct antiviral mechanisms, such as production and release of antimicrobial peptides [7]. Suppression of the type-1 interferon immune response by the viral infection may represent a key immunological mechanism predisposing these patients to develop CAPA [1, 5]. However, severe viral infections may also cause depletion of B1a lymphocytes affecting production of anti-Aspergillus Immunoglobulin G (IgG), and thereby allowing the fungus to remain concealed from recruited lung neutrophils [8, 9]. In addition to the direct effects caused by viral infection, adjunctive immunosuppressive/immunomodulatory treatment for the treatment of moderate to severe COVID-19 may exacerbate the risk of developing CAPA [5, 10].

The overall burden of CAPA in critically ill COVID-19 patients is challenging to assess and wide variability of CAPA prevalence has been reported [11]. In a pan-European multicenter study, inter-center CAPA prevalence rate varied between 1.7 to 26.8%, with a median prevalence of 11% [12]. This variance in incidence rates was also observed among many other studies [5] and has several potential reasons including availability of fungal diagnostics, local epidemiology, demographics and socio-economic factors. High prevalence and mortality rates [13] of CAPA raised the question whether critically ill COVID-19 patients may benefit from application of mold-active antifungal prophylaxis, similar to other high-risk groups [14–17]. Additionally, diagnosis of invasive aspergillosis in the ICU is often challenging, given the often unspecific clinical and radiological presentation as well as the risks associated with invasive diagnostic procedures, even though bronchoscopy is a well-tolerated procedure in ventilated COVID-19 patients [18].

In a recently published observational data obtained from several ICUs from our center, it could be shown, that CAPA prevalence rate may significantly be reduced by application of antifungal prophylaxis [15]. However, antifungal prophylaxis was not associated with survival benefits in critical ill COVID-19 patients. Here we report our local experience with CAPA in a medical ICU, and the impact of systemic mold active prophylaxis in critically ill COVID-19 patients on CAPA development. We report this data in addition to the data reported earlier [15], as we have observed that the baseline characteristics of critically ill COVID-19 patients have changed over the time of the pandemic. We now observe a higher number of immunocompromised patients in the ICU (e.g., active malignancies) compared to the early phases of the pandemic and associated with that, higher rates of CAPA and higher mortality rates [19]. In addition, we aimed to include data on CAPA epidemiology and diagnostic work-up in a cohort of patients receiving antifungal prophylaxis in a homogenous cohort of critically ill COVID-19 patients, which has not been reported before.

## Methods

### Study Cohort

This is a prospective monocentric observational study performed at the University Hospital of Graz, Austria. The primary objective of this study was to describe our local experience with CAPA, and to compare the incidence of CAPA between patients who developed COVID-19 associated respiratory failure (ARF) and who received antifungal prophylaxis versus those who did not receive prophylaxis. Secondary objectives were the comparison of demographic data and outcome between the two groups.

At our institution, systemic antifungal prophylaxis with posaconazole [intravenous (i.v.) or oral tablet formulation] 300 mg twice daily at day 1, followed by 300 mg once daily was recommended for all patients with COVID-19 associated ARF for the duration of respiratory support (non-invasive or invasive) in September 2020. Alternatively, inhaled liposomal amphotericin B (lipAmB) at a dose of 12 mg thrice weekly could have been used in patients who had a contraindication for posaconazole. The decision whether or not to implement antifungal prophylaxis, however, was solely at the treating physician’s discretion and also depended on the time the local recommendations where published.

All consecutive COVID-19 patients who developed ARF and were admitted to our medical ICU between April 2020 and May 2021, were considered eligible for study inclusion. Inclusion criteria were a positive SARS-CoV-2 polymerase chain reaction (PCR) result and admission to ICU due to COVID-19 associated ARF. Exclusion criteria were age < 18 years and other reasons than COVID-19 associated ARF for ICU admission.

All included patients were classified as having proven CAPA, probable CAPA, possible CAPA or no CAPA based on the 2020 ECMM/ISHAM consensus definitions [20].

Part of the data from a part of the study cohort reported here, have been reported in earlier publications [12, 13, 15, 21]. The study was approved by the local ethics committee (32–296 ex 19/20).

### Statistical Analysis

For statistical analysis IBM SPSS 27 (SPSS Inc., Chicago, IL) was used. For the descriptive analysis categorical variables were displayed in absolute and relative frequencies with counts and percentages. Quantitative variables were presented as medians and quartiles or as mean plus 95% confidence interval (95% CI), as appropriate. To compare the group of patients who received antifungal prophylaxis versus those with no antifungal prophylaxis and test for statistical significance, categorical variables were tested with chi-squared test and Fisher’s exact test, respectively. All quantitative variables were analyzed for normal distribution. Quantitative variables were then tested for statistically significant difference between the two groups with Mann–Whitney-u-test or unpaired t-test, as appropriate. Survival curves of patients with and without CAPA are displayed as Kaplan–Meier curve. The impact of CAPA diagnosis on survival status of COVID-19 patients was assessed by the log-rank test. A *p* value < 0.05 was considered statistically significant.

## Results

### Study Cohort

Baseline characteristics of the study cohort are displayed in Table 1. Seventy-seven patients were admitted to the ICU during the observational period, fulfilled inclusion criteria and were enrolled into the study. Twenty-seven patients (35.1%) were female and 50 (64.9%) had a cardiovascular disease as a risk factor for a severe COVID-19 course. Second most prevalent underlying condition was chronic lung disease in 26 patients (33.8%), followed by obesity defined as body-mass-index > 30 (27.3%), diabetes mellitus (23.4%), malignancies (15.6%), and history of smoking (14.3%). Six patients (7.8%) were recipients of a solid organ transplant. Thirty patients (39.0%) patients received non-invasive ventilation only on ICU, and 47 (61.0%) patients required invasive mechanical. Out of the 47 patients who received invasive mechanical ventilation, seven received additional extracorporeal membrane oxygenation (ECMO) treatment.

**Table 1:** Baseline characteristics of study cohort

	Total (*n* = 77)	Prophylaxis group (*n* = 53)	No prophylaxis group (*n* = 24)	*P* value
Age (years), median (25th–75th quartile)	62 (55–72.5)	62 (54–72.5)	61.5 (55–74.3)	n.s
Male sex, n (%)	50 (64.9)	35 (66)	15 (62.5)	n.s
CAPA classification				
Proven	0	0	0	n.a
Probable	6	0	6	< 0.001
Possible	0	0	0	n.a
Baseline characteristics, n (%)				
Cardiovascular disease	50 (64.9)	34 (64.2)	16 (66.7)	n.s
Diabetes mellitus	18 (23.4)	10 (18.9)	8 (33.3)	n.s
History of smoking	11 (14.3)	7 (13.2)	4 (16.7)	n.s
Malignant disease	12 (15.6)	6 (11.3)	6 (25)	n.s
Obesity (BMI > 30 kg/m^2^)	21 (27.3)	17 (32.1)	4 (16.7)	n.s
Pulmonary disease	26 (33.8)	17 (32.1)	9 (37.5)	n.s
Solid organ transplantation	6 (7.8)	5 (9.4)	1 (4.2)	n.s
Respiratory support on ICU, n (%)				
Non-invasive ventilation	30 (39.0)	19 (35.8)	11 (45.8)	n.s
Invasive mechanical ventilation	47 (61.0)	34 (64.2)	13 (54.2)	n.s
ECMO*	7 (5.2)	7 (13.2)	0 (0)	n.s
COVID-19 therapy, n (%)				
Systemic corticosteroids	63 (81.8)	46 (86.8)	17 (70.8)	n.s
Tocilizumab	5 (6.5)	1 (1.9)	4 (16.7)	0.031
Outcome				
Survival day 30, n (%)	49 (63.6)	30 (56.6)	19 (79.2)	n.s
Survival at ICU discharge, n (%)	43 (55.8)	28 (52.8)	15 (62.5)	n.s
ICU stay (days), median (25th–75th quartile)	11 (5–23)	10 (5–20)	12.5 (4.5–33.75)	n.s

*BMI* = Body Mass Index, *CAPA* = COVID associated pulmonary aspergillosis, *ECMO* = Extracorporeal membrane oxygenation; *EORTC* = European Organization for Research and Treatment of Cancer, *ICU* = Intensive Care Unit; n.s. = not significant (*p* ≥ 0.05)

^*^All patients with ECMO had invasive mechanical ventilation at time of ECMO initiation and were counted as “invasive mechanical ventilation” and “ECMO”

### Antifungal Prophylaxis, CAPA Development and Outcome

Fifty-three patients (68.8%) received antifungal prophylaxis during their ICU stay as part of their routine management. Posaconazole was used as prophylaxis in all 53 patients, and all patients received posaconazole intravenously. As none of the patients had contraindications against the routine use of posaconazole, inhaled lipAmB was not used in this cohort during the observational period.

In the total study cohort, six patients were routinely diagnosed with CAPA. All CAPA cases were classified as probable CAPA. CAPA was only diagnosed in the non-prophylaxis group with six cases versus no case in the prophylaxis group (p < 0.001). CAPA was diagnosed at a median of 8.5 days (25–75th: 3–18.25) following ICU admission. The incidence of CAPA in the overall cohort was 0.57 events per 100 ICU days (95% CI 0.53–0.62) and 2.2 events per 100 ICU days (95% CI 2.02–2.37) in the non-prophylaxis group.

The median length stay in the ICU was 10 days (25–75th: 5–20) in the non-CAPA group versus 36.5 days (25–75th: 33–42.25) in the group of patients diagnosed with CAPA (p < 0.001). Median observation time from ICU admission to last follow-up visit for the whole study cohort was 30 days (25–75th: 15–39.5). For patients who were diagnosed with CAPA, the median follow-up time after CAPA diagnosis was 32 days (25–75th: 24.75–49.5). Thirty days after ICU admission 30 patients (56.5%) in the prophylaxis group were still alive, while 19 patients (79.2%) in the non-prophylaxis group were still alive. At ICU discharge 28 patients (52.8%) in the prophylaxis group were still alive, while 15 patients (62.5%) in the non-prophylaxis group were still alive. Out of the six CAPA patients, four were discharged alive from ICU (66.6%). In the group of non-CAPA patients, 39 out of 71 patients (54.9%) were discharged alive (*p* = 0.689).

**Fig. 1:**
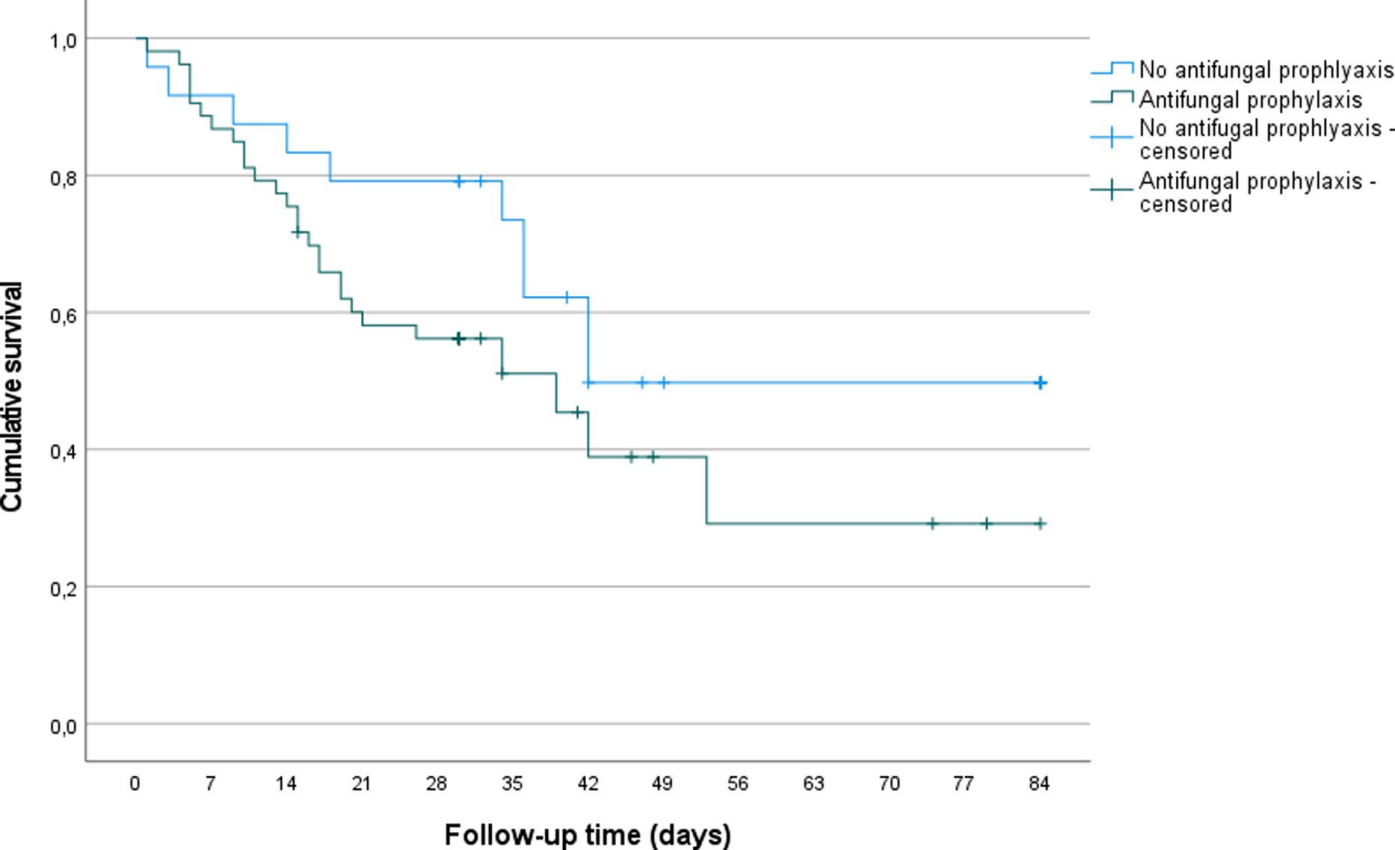
Kaplan–Meier survival curves between patients who received antifungal prophylaxis and those you did not for an 84-day follow-up period. No differences in probability of survival could be observed between the groups (Log-rank test: *p* = 0.115)

No difference in the cumulative 84-day survival for individuals who received antifungal prophylaxis versus those who did not could be observed (Fig. 1; *p* = 0.115). Median survival time was estimated with 42 days (95% CI 30.24–53.76) in the CAPA group and 42 days (95% CI 33.29–50.71) in the group of patients without developing CAPA (*p* > 0.05).

Administration of systemic glucocorticosteroids did not differ significantly between both groups. Forty-six patients (86.8%) in the prophylaxis group received glucocorticosteroids versus 17 patients (70.8%) in the control group. In contrast, tocilizumab was significantly more often applied in the non-prophylaxis group with four patients versus one patient in the prophylaxis group (*p* = 0.031).

### CAPA Diagnosis

Bronchoscopy including BALF-GM testing was performed in 11 out of the 24 patients (45%) that did not receive antifungal prophylaxis and in 19 out of the 53 patients (36%) who received antifungal prophylaxis (*p* > 0.05). In addition, baseline characteristic (e.g., immunosuppressive disease) were similar among the CAPA and non-CAPA group. All six CAPA cases had BALF-GM testing. In the group of patients without CAPA, BALF-GM was performed in 24 out of 71 patients (34%).

In four out of the six CAPA patients, there was a positive BALF-GM result with an optical density index (ODI) > 1. Two patients had a positive GM result in serum with an ODI > 0.5. Four out of six patients had a positive *Aspergillus* spp. specific polymerase chain reaction (PCR) result in BALF. There was one positive *Aspergillus* spp. lateral flow device (LFD) result in BALF in the total cohort (this patient also had a positive BALF-GM and BALF-Aspergillus PCR), one positive *Aspergillus* spp. culture result in BALF and one positive *Aspergillus* spp. culture result in tracheal aspirate.

## Discussion

In this prospective monocentric cohort study in critically ill COVID-19 patients, we observed a CAPA incidence of 2.2 events per 100 ICU days in those not receiving antifungal prophylaxis, while not a single case of CAPA was observed in those with administration of mold active antifungal prophylaxis. No impact on cumulative 84 days survival, however, was observed.

Antifungal prophylaxis has been shown to significantly reduce the incidence of invasive fungal disease (IFD) in different cohorts of patients with hematological malignancies who are considered to be at high risk for IFD development [22–25]. Prevalence rates of IFDs of up to 25% have been reported in neutropenic patients in the pre-prophylaxis era [26]. Similar rates have been observed in other cohorts of critically ill influenza and COVID-19 patients [2, 10, 12, 27]. To evaluate the impact of antifungal prophylaxis on IAPA, the randomized POSA-FLU trial has been conducted. This study investigated the safety and efficacy of posaconazole prophylaxis in critically ill influenza patients [28]. The trial showed a trend towards reduced incidence of IAPA in the posaconazole arm versus the placebo arm (5.4% versus 11.1%), but failed to prove statistical significance, because of lack of power and due to the fact that the rate of early IAPA after ICU admission (within 48 h) was higher than expected. In addition, prophylaxis was limited to a maximum of seven days, however, two cases of IAPA were diagnosed after day 7 (day 8 and 12, respectively). In contrast to this finding, we observed, that the time from ICU admission to CAPA diagnosis was longer in the cohort reported here (median 8.5 days). This is in concordance with reports from other studies [5]. The longer period may allow antifungal prophylaxis to reduce the rate of fungal infections more significantly compared to influenza, as mean *C*_min_ levels of > 1000 mg/L are achieved approximately 3 days after start of intravenous posaconazole [29]. Even though, it may take several more days to reach a solid steady state (approximately 7 days), this would be sufficient to achieve solid trough levels before the majority of CAPA cases are clinically diagnosed. Also, in patients who receive ECMO, posaconazole plasma concentrations of ≥ 1 mg/L will be reached in the majority of patients within 48 h [30]. Concordant with this, we observed a significantly reduced CAPA incidence rate in the prophylaxis group. In line with another larger study from our center, evaluating a shorter timeframe but involving multiple ICUs across our hospital [15], we could not observe a significant reduction in mortality in the group of patients who received antifungal prophylaxis. This observation may be, at least partly, explained by the fact that our study was neither designed, nor aimed to detect a difference in mortality. In addition, all patients who needed ECMO treatment in our cohort received antifungal prophylaxis, whereas no patient in the non-prophylaxis group was treated with ECMO. Taken together with the finding, that 30-days after ICU admission, more patients in the non-prophylaxis group were alive compared to the prophylaxis group, one may hypothesize, that patients in the prophylaxis group may had more severe disease and a poorer prognosis, regardless of the application of antifungal prophylaxis. In addition, the fact that there was no survival benefit for the prophylaxis group may, at least partly, be explained by the pathophysiological hallmarks of CAPA. In contrast to invasive aspergillosis in severely neutropenic patients and also observed in critically ill influenza patients, CAPA does not primarily cause angio-invasive infection [31]. Angio-invasion in CAPA is usually observed at later stages of the disease. In general, we observed that CAPA diagnosis is made at a median of 8.5 days after ICU admission. This implicates, that patients who die earlier from COVID-19 in ICUs cannot develop CAPA. This is potentially causing a bias towards a higher mortality rate in the non-CAPA cohort. As all CAPA cases occurred in the non-prophylaxis group, this, however, may partly explain the fact that we could not observe a significant survival benefit between the two groups.

Thereby Kaplan–Meier analyses starting at the day of ICU admission are biased by the fact that the control arm includes all those who are more severely ill and have a fatal outcome before they can develop CAPA. In contrast, multiple large studies have shown that mycological evidence for CAPA per se, like positive BAL GM or BAL culture and especially positive serum GM are associated with significantly higher mortality rates often exceeding 80% [21, 31, 32].

Different reasons may explain the pathophysiological differences between IAPA and CAPA, including the lack of neutropenia in many patients with COVID-19 in ICUs as well as the different pathophysiology of COVID-19 compared to influenza. Infection with influenza virus, for example, does cause severe lytic infections in the respiratory epithelium and therefore mitigate early invasive growth of Aspergillus [6]. In addition, influenza has shown to affect some defense mechanisms against pulmonary infections like the NADPH-depended production of reactive oxygen species in macrophages and neutrophils [33]. However, application of immunomodulatory drugs including glucocorticosteroids or anti-IL-6 treatment is not standard of care for severe influenza but for severe COVID-19, which may contribute to the elevated risk of pulmonary aspergillosis in critically ill COVID-19 patients. In our cohort, there was only a relatively small number (*N* = 5) of subjects that received tocilizumab, however, majority of these patients (*n* = 4) were in the non-prophylaxis group. As tocilizumab treatment is considered an independent risk factor for the development of CAPA [12], this may partly contributed to the higher CAPA incidence in the non-prophylaxis group in this study. In general, we do not exactly know the reasons why antifungal prophylaxis was withhold in some patients. One may only speculate that, especially in the early phase of the pandemic, the burden of CAPA and risk factors for CAPA development had not been clearly identified. For some physicians, the risk–benefit ratio may therefore be difficult to establish, considering the potential side effects of antifungals in the ICU and the unclear benefit–especially in terms of overall outcome. Some physicians therefore may have favored a pre-emptive strategy, even though we know now, that screening for CAPA is difficult based on the limited sensitivity of blood biomarkers and the need for invasive procedures like bronchoscopies.

In this study, BALF-GM was the main mycological diagnostic criterium, however, also serum-GM turned out positive in two out of the six CAPA patients indicating angio-invasive disease. Next generation sequencing of plasma samples may overcome some of the limitations of conventional blood GM testing and showed promising results for CAPA diagnosis in a subgroup of CAPA patients reported here [34].

The influence of different SARS-CoV-2 strains on the epidemiology of CAPA and consequently on the management strategies, including antifungal prophylaxis, is not fully understood yet. CAPA incidence rates may also differ with the predominant SARS-CoV-2 strains [35], as observed for influenza [36]. In this study, we covered several COVID-19 waves and observed CAPA cases in all of them, including one CAPA case that was diagnosed in the period (before September 2020) where there was no local recommendation for antifungal prophylaxis in critically ill COVID-19 patients. Whether future variants of SARS-CoV-2 or adaptions in the COVID-19 management will affect the epidemiology of CAPA needs to be closely observed, as this may also affect the strategies for CAPA management and prevention.

Based on currently available data, no recommendation can be given for or against the general use of antifungal prophylaxis in critically ill COVID-19 patients. This is also influenced by several factors like the wide variation on local epidemiology, the use of (combination) immunomodulatory treatment, individual risk factors like underlying immunosuppressive disease, potential transient risk factor like construction work, and draw backs of azole usage in the ICU like drug-drug interactions or toxicities. Besides antifungal prophylaxis, however, fungal awareness is key for early diagnosis and treatment. This is critical, as it is well-known, that true fungal infections in COVID-19 patients are associated with reduced probability of survival [12, 37].

This report highlighted the local experience with application of antifungal prophylaxis in critically ill COVID-19 patients. As this was a non-interventional observational trial, it does come with some important limitations that should be considered: First, the uncontrolled study design does not allow for equal distribution of risk factors for CAPA development or poor outcome among the two groups. As some variables including APACHE-II score, SOFA score or details on EORTC/MSGERC risk factors for fungal infections were not available, it cannot be excluded, that baseline characteristics were distributed unequally among the two groups. Second, as no systemic antifungal screening protocol was implemented at our center, the decision whether or not to perform antifungal diagnostics was solely based on the treating physician’s discretion. This may cause over- or underdiagnoses of CAPA in one of the groups. Third, we did not observe a biopsy proven CAPA case, which is due to the fact, that lung biopsy in critically ill COVID-19 patients is usually not possible. Lastly, the study was not designed to investigate a survival benefit of antifungal prophylaxis in this cohort. In addition, a cox regression model couldn’t be performed to implement CAPA as a time-dependent variable and avoid immortality bias due to the small sample size of CAPA cases and not fulfilling the assumption of proportional hazards.

In conclusion, in this observational cohort we found that the application of mold active antifungal prophylaxis in critically ill COVID-19 patients was associated with significantly reduced number of CAPA cases, while we could not observe an effect on overall survival.

### Supplementary Information

Below is the link to the electronic supplementary material.

Supplementary file1 (DOCX 13 KB)

## References

[1] Feys S, Gonçalves SM, Khan M, Choi S, Boeckx B, Chatelain D (2022). Lung epithelial and myeloid innate immunity in influenza-associated or COVID-19-associated pulmonary aspergillosis: an observational study. Lancet Respir Med..

[2] Schauwvlieghe AFAD, Rijnders BJA, Philips N, Verwijs R, Vanderbeke L, Van Tienen C (2018). Invasive aspergillosis in patients admitted to the intensive care unit with severe influenza: a retrospective cohort study. Lancet Respir Med..

[3] Huang C, Wang Y, Li X, Ren L, Zhao J, Hu Y (2020). Clinical features of patients infected with 2019 novel coronavirus in Wuhan. China Lancet..

[4] Yang X, Yu Y, Xu J, Shu H, Xia J, Liu H (2020). Clinical course and outcomes of critically ill patients with SARS-CoV-2 pneumonia in Wuhan, China: a single-centered, retrospective, observational study. Lancet Respir Med..

[5] Hoenigl M, Seidel D, Sprute R, Cunha C, Oliverio M, Goldman GH (2022). COVID-19-associated fungal infections. Nat Microbiol..

[6] Dewi IM, Janssen NA, Rosati D, Bruno M, Netea MG, Brüggemann RJ (2021). Invasive pulmonary aspergillosis associated with viral pneumonitis. Curr Opin Microbiol..

[7] Chua RL, Lukassen S, Trump S, Hennig BP, Wendisch D, Pott F (2020). COVID-19 severity correlates with airway epithelium–immune cell interactions identified by single-cell analysis. Nat Biotechnol..

[8] Sarden N, Sinha S, Potts KG, Pernet E, Hiroki CH, Hassanabad MF (2022). A B1a–natural IgG–neutrophil axis is impaired in viral- and steroid-associated aspergillosis. Sci Transl Med..

[9] Gangneux JP, Hoenigl M, Papon N (2023). How to lose resistance to aspergillus infections. Trends Microbiol..

[10] Gangneux JP, Dannaoui E, Fekkar A, Luyt CE, Botterel F, De Prost N (2022). Fungal infections in mechanically ventilated patients with COVID-19 during the first wave: the French multicentre MYCOVID study. Lancet Respir Med..

[11] Egger M, Bussini L, Hoenigl M, Bartoletti M (2022). Prevalence of COVID-19-associated pulmonary aspergillosis: critical review and conclusions. J Fungi..

[12] Prattes J, Wauters J, Giacobbe DR, Salmanton-García J, Maertens J, Bourgeois M (2021). Risk factors and outcome of pulmonary aspergillosis in critically ill coronavirus disease 2019 patients—a multinational observational study by the European confederation of medical mycology. Clin Microbiol Infect..

[13] Prattes J, Wauters J, Giacobbe DR, Lagrou K, Hoenigl M (2021). Diagnosis and treatment of COVID-19 associated pulmonary apergillosis in critically ill patients: results from a European confederation of medical mycology registry. Intensive Care Med..

[14] Verweij PE, Brüggemann RJM, Azoulay E, Bassetti M, Blot S, Buil JB (2021). Taskforce report on the diagnosis and clinical management of COVID-19 associated pulmonary aspergillosis. Intensive Care Med..

[15] Hatzl S, Reisinger AC, Posch F, Prattes J, Stradner M, Pilz S (2021). Antifungal prophylaxis for prevention of COVID-19-associated pulmonary aspergillosis in critically ill patients: an observational study. Crit Care.

[16] Melchers M, van Zanten ARH, Heusinkveld M, Leeuwis JW, Schellaars R, Lammers HJW (2022). Nebulized amphotericin b in mechanically ventilated COVID-19 patients to prevent invasive pulmonary aspergillosis: a retrospective cohort study. Crit Care Explor.

[17] Van Ackerbroeck S, Rutsaert L, Roelant E, Dillen K, Wauters J, Van Regenmortel N (2021). Inhaled liposomal amphotericin-B as a prophylactic treatment for COVID-19-associated pulmonary aspergillosis/aspergillus tracheobronchitis. Crit Care.

[18] Saha BK, Saha S, Chong WH, Beegle S (2022). Indications, clinical utility, and safety of bronchoscopy in COVID-19. Respir Care.

[19] Feys S, Lagrou K, Lauwers HM, Haenen K, Jacobs C, Brusselmans M (2023). High burden of COVID-19-associated pulmonary aspergillosis (CAPA) in severely immunocompromised patients requiring mechanical ventilation. Clin Infect Dis..

[20] Koehler P, Bassetti M, Chakrabarti A, Chen SCA, Colombo AL, Hoenigl M (2020). Defining and managing COVID-19-associated pulmonary aspergillosis: the 2020 ECMM/ISHAM consensus criteria for research and clinical guidance. Lancet Infect Dis..

[21] Giacobbe DR, Prattes J, Wauters J, Dettori S, Signori A, Salmanton-García J (2022). Prognostic impact of bronchoalveolar lavage fluid galactomannan and aspergillus culture results on survival in COVID-19 intensive care unit patients: a post hoc analysis from the european confederation of medical mycology (ECMM) COVID-19-associated pulmonary aspergillosis study. J Clin Microbiol..

[22] Cornely OA, Perfect J, Helfgott D, Goh YT, Suresh R (2007). Posaconazole vs. fluconazole or itraconazole prophylaxis in patients with neutropenia. N Engl J Med..

[23] Ullmann AJ, Langston A, Reddy V (2007). Posaconazole or fluconazole for prophylaxis in severe graft-versus-host disease. N Engl J Med..

[24] Rotstein C, Bow EJ, Laverdiere M, Ioannou S, Carr D, Moghaddam N (1999). Randomized placebo-controlled trial of fluconazole prophylaxis for neutropenic cancer patients: benefit based on purpose and intensity of cytotoxic therapy. Clin Infect Dis..

[25] Wingard JR, Carter SL, Walsh TJ, Kurtzberg J, Small TN, Baden LR (2010). Randomized, double-blind trial of fluconazole versus voriconazole for prevention of invasive fungal infection after allogeneic hematopoietic cell transplantation. Blood.

[26] Menichetti F, Del Favero A, Martino P, Bucaneve G, Micozzi A, Girmenia C (1999). Itraconazole oral solution as prophylaxis for fungal infections in neutropenic patients with hematologic malignancies: a randomized, placebo-controlled, double-blind. Multicenter Trial Clin Infect Dis..

[27] Nyga R, Maizel J, Nseir S, Chouaki T, Milic I, Roger PA (2020). Invasive tracheobronchial aspergillosis in critically ill patients with severe influenza. A Clinical Trial Am J Respir Crit Care Med..

[28] Vanderbeke L, Janssen NAF, Bergmans DCJJ, Bourgeois M, Buil JB, Debaveye Y (2021). Posaconazole for prevention of invasive pulmonary aspergillosis in critically ill influenza patients (POSA-FLU): a randomised, open-label, proof-of-concept trial. Intensive Care Med..

[29] Cornely OA, Robertson MN, Haider S, Grigg A, Geddes M, Aoun M (2017). Pharmacokinetics and safety results from the phase 3 randomized, open-label, study of intravenous posaconazole in patients at risk of invasive fungal disease. J Antimicrob Chemother..

[30] Van Daele R, Brüggemann RJ, Dreesen E, Depuydt P, Rijnders B, Cotton F (2021). Pharmacokinetics and target attainment of intravenous posaconazole in critically ill patients during extracorporeal membrane oxygenation. J Antimicrob Chemother..

[31] Ergün M, Brüggemann RJM, Alanio A, Dellière S, van Arkel A, Bentvelsen RG (2021). Aspergillus test profiles and mortality in critically Ill COVID-19 patients. J Clin Microbiol..

[32] Dellière S, Dudoignon E, Voicu S, Collet M, Fodil S, Plaud B (2022). Combination of mycological criteria: a better surrogate to identify COVID-19-associated pulmonary aspergillosis patients and evaluate prognosis?. J Clin Microbiol..

[33] Sun K, Metzger DW (2014). Influenza infection suppresses NADPH oxidase-dependent phagocytic bacterial clearance and enhances susceptibility to secondary methicillin-resistant *staphylococcus aureus* infection. J Immunol..

[34] Hoenigl M, Egger M, Price J, Krause R, Prattes J, White PL (2023). Metagenomic next-generation sequencing of plasma for diagnosis of COVID-19-associated pulmonary aspergillosis. J Clin Microbiol..

[35] Meijer EFJ, Dofferhoff ASM, Hoiting O, Meis JF (2021). COVID-19–associated pulmonary aspergillosis: a prospective single-center dual case series. Mycoses..

[36] Schwartz IS, Friedman DZP, Zapernick L, Dingle TC, Lee N, Sligl W (2020). High rates of influenza-associated invasive pulmonary aspergillosis may not be universal: a retrospective cohort study from Alberta. Canada Clin Infect Dis..

[37] Verweij PE, Van De Veerdonk FL (2022). Managing secondary fungal infections in severe COVID-19: how to move forward?. Lancet Respir Med..

