# Supplementary material for: COVID-19 associated Pulmonary Aspergillosis in Patients Admitted to the Intensive Care Unit: Impact of Antifungal Prophylaxis
Source: Mycopathologia. 2024 Jan 13;189(1):3. doi: 10.1007/s11046-023-00809-y (PMC10787678; doi:10.1007/s11046-023-00809-y)
Supplement: Supplementary file 1 — Supplementary file1 (DOCX 13 KB) [file 11046_2023_809_MOESM1_ESM.docx]

**Supplementary Table S1: Mycological criteria for CAPA patients**

|  | **BALF** | | | | **Blood** |
| --- | --- | --- | --- | --- | --- |
| **Subject** | **GM** | **Aspergillus culture** | **Aspergillus PCR** | **Aspergillus specific LFD** | **GM** |
| **CAPA 01** | - | - | N.D | - | + |
| **CAPA 02** | + | - | + | N.D. | - |
| **CAPA 03** | - | - | + | N.D. | - |
| **CAPA 04** | + | - | + | N.D. | - |
| **CAPA 05** | + | + | + | + | + |
| **CAPA 06** | + | - | N.D. | N.D. | - |

Abbrevations: BALF = bronchoalveolar lavage fluid; N.D. = not done

Results of mycological testing for CAPA (n=6) according to the 2020 ECMM/ISHAM consensus criteria (Koehler P. et al.; DOI: 10.1016/ S1473-3099(20)30847-1)
